# Fermented *Taiwanofungus camphoratus* Extract Ameliorates Psoriasis-Associated Response in HaCaT Cells via Modulating NF-𝜅B and mTOR Pathways

**DOI:** 10.3390/ijms232314623

**Published:** 2022-11-23

**Authors:** Jia-Wei Shen, Po-Yuan Wu, Yueh-Hsiung Kuo, Qiao-Xin Chang, Kuo-Ching Wen, Hsiu-Mei Chiang

**Affiliations:** 1 Department of Cosmeceutics, China Medical University, Taichung 406, Taiwan; 2 Department of Dermatology, China Medical University Hospital, Taichung 404, Taiwan; 3 School of Medicine, China Medical University, Taichung 404, Taiwan; 4 Department of Chinese Pharmaceutical Sciences and Chinese Medicine Resources, China Medical University, Taichung 404, Taiwan; 5 Department of Biotechnology, Asia University, Taichung 413, Taiwan; 6 Ph.D. Program for Biotechnology Industry, School of Life Sciences, China Medical University, Taichung 406, Taiwan

**Keywords:** *Taiwanofungus camphoratus*, psoriasis, IL-17A, nuclear factor kappa B, mammalian target of rapamycin

## Abstract

Psoriasis is a chronic autoimmune disease, and until now, it remains an incurable disease. Therefore, the development of new drugs or agents that ameliorate the disease will have marketing potential. *Taiwanofungus camphoratus* (TC) is a specific fungus in Taiwan. It is demonstrated to have anticancer, anti-inflammation, and hepatoprotective effects. However, the effects of TC fermented extract on psoriasis are under investigation. In this research, we studied the ability of TC on antioxidative activity and the efficacy of TC on interleukin-17 (IL-17A)-induced intracellular oxidative stress, inflammation-relative, and proliferation-relative protein expression in human keratinocytes. The results of a DPPH radical scavenging assay, reducing power assay, and hydroxyl peroxide inhibition assay indicated that TC has a potent antioxidant ability. Furthermore, TC could reduce IL-17A-induced intracellular ROS generation and restore the NADPH level. In the investigation of pathogenesis, we discovered TC could regulate inflammatory and cell proliferation pathways via *p*-IKKα/*p*-p65 and *p*-mTOR/*p*-p70S6k signaling pathways in human keratinocytes. In conclusion, TC showed characteristics such as antioxidant, anti-inflammatory, and anti-psoriatic-associated responses. It is expected to be developed as a candidate for oxidative-stress-induced skin disorders or psoriasis treatment.

## 1. Introduction

Psoriasis is a chronic autoimmune disease [1]. The worldwide prevalence is approximately 2–3% of the total population [2]. Unfortunately, it is an incurable disease at this time. The characteristics of psoriatic skin lesions involve erythema, desquamation, and induration. Histological characteristics of the disease reveal that sustained inflammation affects aberrant cell proliferation of keratinocytes and causes immune infiltration [3]. The inflammatory cascade secretes cytokines such as tumor necrosis factor-α (TNF-α), interleukin-6 (IL-6), and interferon gamma (IFNγ), released by keratinocytes, dendritic cells, and macrophages [4]. These cytokines may drive T cell differentiation into Th17 and Th1 cells releasing cytokines, including IL-17A, IL-17F, and IL-2. The interplay between keratinocytes and lymphocytes develops inflammation and immune response, resulting in psoriasis generation [5].

Oxidative stress plays an important role in cellular biological and pathological processes including cancer, aging, and skin disorders [6]. It was reported that oxidative stress or ROS generation is a major factor causing symptoms and related complications of psoriasis [7]. The levels of oxidative stress markers such as peroxide, catalase, and 8-hydroxy-2′-deoxy-guanosine are related to the severity of psoriasis [8]. Reactive oxygen species (ROS) generation triggers several signaling pathways related to psoriasis including mitogen-activated protein kinase (MAPK), activator protein 1 (AP-1), nuclear factor kappa B (NF-kB), and Janus kinase-signal transducer and activator of transcription (JAK-STAT) [7]. Therefore, materials or natural products with antioxidative activity may ameliorate the symptoms of psoriasis.

The activation of the inflammatory pathway by IL-17 in keratinocytes involves MAPK, signal transducer and activator of transcription 1 (STAT1), and NF-κB [9]. After combining with the receptor, IL-17 induces cell signaling to form the complex of NF-κB and IκB kinase (IKK), stimulating phosphorylation of IκB and translocating subunits of NF-κB such as p65 and p50 into the nucleus. The activation of the NF-κB pathway generates inflammatory-related proteins and cytokines such as IL-6, IL-8, IL-1, and TNF-α, and these effectors can up-regulate signaling pathways, sustaining an inflammatory response [10]. IL-17A binding with receptor IL-17RA induces NADPH oxidase-dependent ROS, dysregulating pathways such as NF-κB, and the mammalian target of rapamycin (mTOR) pathway, resulting in inflammation and cell hyperplasia conditions [11,12]. In addition, the inhibition of IL-17A attenuates the ROS level in the psoriasis-like mouse model blood sample [13].

The protein kinase B (Akt)/mammalian target of rapamycin (mTOR)/p70 ribosomal protein s6 kinase (p70S6K) signaling pathway is involved with cell proliferation, invasion, and motility [14]. The phosphorylation of Akt activates downstream molecules such as mTOR. Subsequently, mTOR increases p70 ribosomal protein s6 kinase phosphorylation (*p*-p70S6K) and inhibits the activation of 4E-binding protein 1 (4E-BP1), causing protein synthesis resulting in cell proliferation [15]. Since psoriasis is associated with the hyper-proliferation of keratinocytes, growing studies demonstrated the mTOR pathway is related to the occurrence of psoriasis [16,17,18].

*Taiwanofungus camphoratus* (TC; *syn. Ganoderma comphoratum*; *Antrodia camphorate*; *Antrodia cinnamomea*) is an endemic medicinal mushroom in Taiwan. The main compounds of TC are polysaccharides and triterpenoids [19]. Reports have investigated the benefits of TC related to anticancer, antioxidant, and hepatoprotective abilities [20,21,22]. In the study of the anti-hepatitis B virus, the TC mycelium exhibited efficacious antiviral properties [23]. The fermented filtrate from TC was shown from the perspective of hepatoprotection in tetrachloromethane-induced hepatic damage rats [24]. The methanol extract of TC protects against acute lung injury by suppressing NF-κB and MAPK pathways [25]. Ergostatrien-3β-ol (EK100), a bioactive component derived from *Taiwanofungus camphoratus*, was demonstrated to improve ultraviolet-induced inflammation and photodamage on hairless mouse skin [26]. Additionally, EK100 inhibited pro-inflammatory cytokine in macrophages [27] and increased the activities of catalase, superoxide dismutase, and glutathione peroxidase to decrease inflammation in the liver of mice [28]. However, reports on the anti-psoriatic function of TC’s mycelia are rare, and the mechanisms need further investigation. In this study, we evaluated the mycelia of TC’s antioxidant ability and studied the IL-17A-induced inflammation and cell proliferation signaling pathways in human keratinocytes (HaCaT cells).

## 2. Results

### 2.1. Quantitation of Bioactive Components in TC

**Figure 1:**
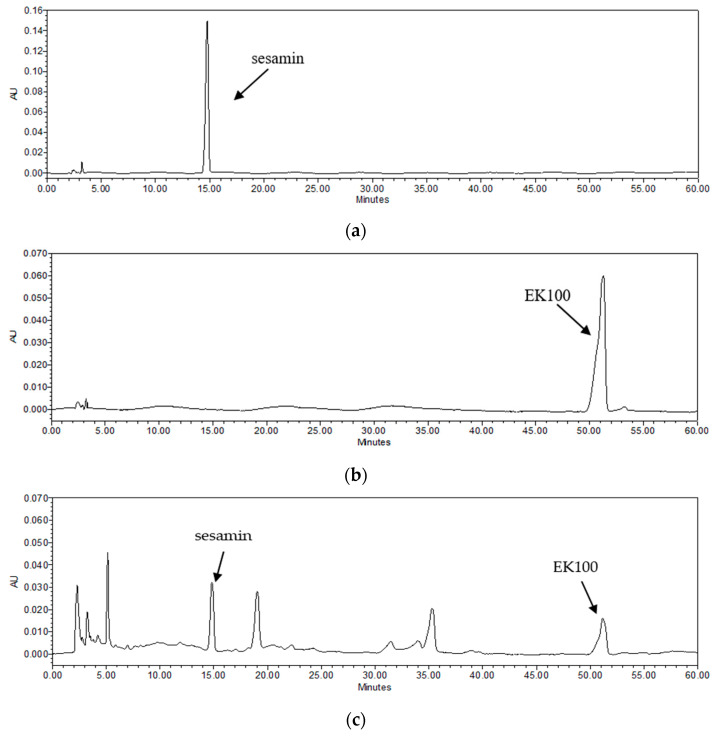
HPLC chromatograms of sesamin and EK100 in TC and standards. (**a**) sesamin standard, (**b**) EK100 standard, and (**c**) sesamin and EK100 in TC.

Plant oils are usually used as natural antifoam agents in fermentation; they are reported to stimulate mycelial growth and to increase bioactive component production [29,30]. TC mycelium and sesame oil undergo co-fermented cultivation in our study. Sesamin is the major lignan in sesame oil [31] and EK100 is an active compound isolated from TC [28]. The analysis results of the active components in TC are shown in Figure 1. The retention times of sesamin and Ergostatrien-3β-ol (EK100) were 14.7 and 51.3 min, respectively. The content of sesamin in TC was 0.25% sesamin and EK100 was 0.30%.

### 2.2. Evaluation of the Antioxidant Ability of TC

#### 2.2.1. DPPH Radical Scavenging Activity

**Figure 2:**
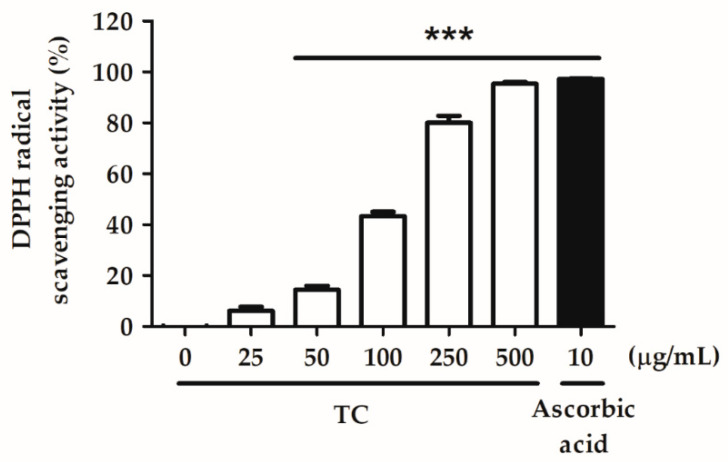
TC scavenges the DPPH radical in a dose-dependent manner. TC exhibited a dose-dependent DPPH radical scavenging ability, with an EC_50_ value of 205.7 µg/mL. Ascorbic acid was used as a positive control. Significant differences between the treated groups and the negative control were evaluated. *** *p* < 0.001 compared to the control group.

Various concentrations of TC (25, 50, 100, 250, and 500 µg/mL) were tested for their ability to scavenge DPPH. Methanol was used as a negative control and ascorbic acid was used as a positive control. The effect concentration (EC_50_) value of TC in the DPPH scavenging assay was 205.7 µg/mL, and 500 µg/mL of TC showed similar efficacy to the positive control (Figure 2). These results indicate that TC is a potent antioxidant.

#### 2.2.2. Reducing Power

**Figure 3:**
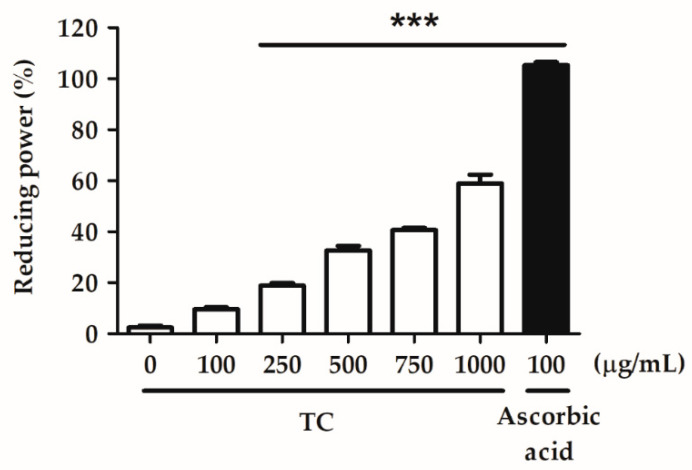
TC displays a concentration-dependent reducing power. TC exhibited a dose-dependent reducing power with an EC_50_ value of 857.9 µg/mL. Ascorbic acid was used as a positive control. Significant differences between the treated groups and the negative control were evaluated. *** *p* < 0.001 compared to the control group.

Various concentrations of TC (100, 250, 500, 750, and 1000 µg/mL) were examined for their ability to reduce the oxidative potential of the test solution. Sterilized water was used as a negative control, and ascorbic acid was used as a positive control. The EC_50_ value of TC in the reducing power assay was 857.9 µg/mL, and the reducing power of TC at concentrations above 250 μg/mL differed significantly from that of the negative control (Figure 3). This result suggests that TC exhibits a remarkable reducing power.

#### 2.2.3. H_2_O_2_ Inhibition Activity

**Figure 4:**
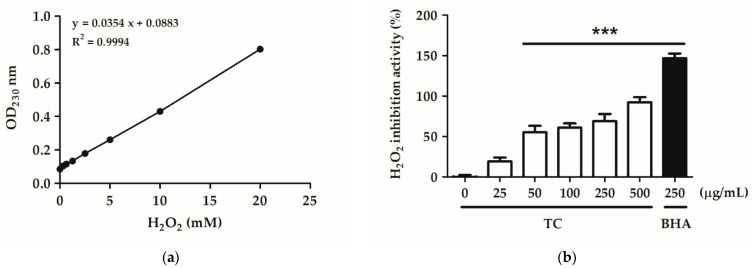
TC scavenges H_2_O_2_ in a dose-dependent manner. (**a**) The standard curve of absorbance at 230 nm at different hydrogen peroxide concentrations. (**b**) The effect of TC on H_2_O_2_ inhibition. TC showed a dose-dependent effect on H_2_O_2_ inhibition, with an EC_50_ value of 488.1 µg/mL and BHA as a positive control. Significant differences between treated groups and the negative control were evaluated. *** *p* < 0.001 compared to the control group.

Various concentrations of TC (25, 50, 100, 250, and 500 µg/mL) were tested for their ability to scavenge H_2_O_2_. PBS buffer was used as a negative control and butylated hydroxyanisole (BHA) as a positive control. The EC_50_ value of TC in the H_2_O_2_ inhibition assay was 488.1 µg/mL, and the H_2_O_2_ inhibition activity of TC at concentrations above 500 µg/mL differed significantly from that of the control group (Figure 4). This result suggests that TC can scavenge H_2_O_2_ in a dose-dependent manner.

### 2.3. Effect of TC on Cell Viability

**Figure 5:**
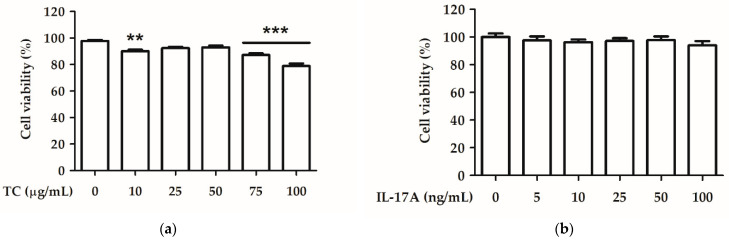
Effect of (**a**) TC and (**b**) IL-17A on cell viability in human keratinocytes. Significant differences between treated groups were evaluated. ** *p* < 0.01; *** *p* < 0.001 compared to the control group.

HaCaT cells were treated with various concentrations of TC solution (10, 25, 50, 75, and 100 µg/mL) for 24 h, and cell viability was analyzed using the MTT assay. A TC concentration of 10–75 µg/mL led to cell viability above 80%, indicating little cell toxicity of TC in human keratinocytes (Figure 5a). HaCaT cells were treated with various concentrations of an IL-17A solution (5, 10, 20, 50, and 100 ng/mL) for 24 h. Figure 5b shows the effect of 5–100 ng/mL IL-17A on cell viability, which was above 80% in all treatments. Therefore, a TC concentration of 10–75 µg/mL and an IL-17A concentration of 50 ng/mL were applied in the following in vitro experiments.

**Figure 6:**
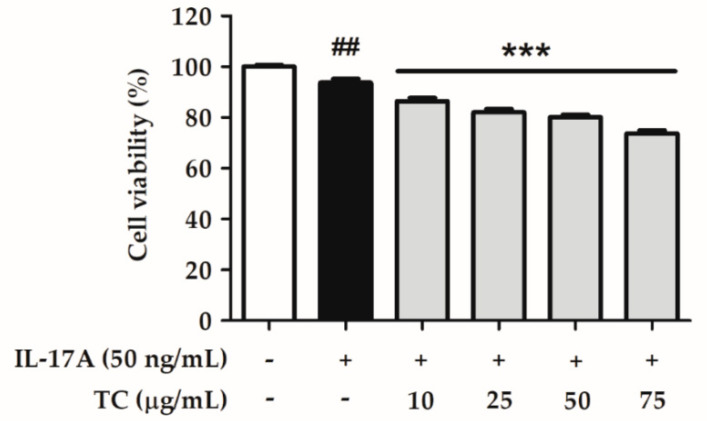
Effect of TC on cell viability in IL-17A-induced human keratinocytes. Significant differences between treated groups were evaluated. **^##^** *p* < 0.01 compared to the control group. *** *p* < 0.001 compared to the IL-17A-induced group.

HaCaT cells were treated with IL-17A, and the cell viability was determined to evaluate the anti-proliferation effect of TC. The results showed that TC (10–75 µg/mL) treatment reduced cell proliferation in HaCaT cells in a dose-dependent manner (Figure 6).

### 2.4. Effect of TC on Intracellular Oxidative Stress

**Figure 7:**
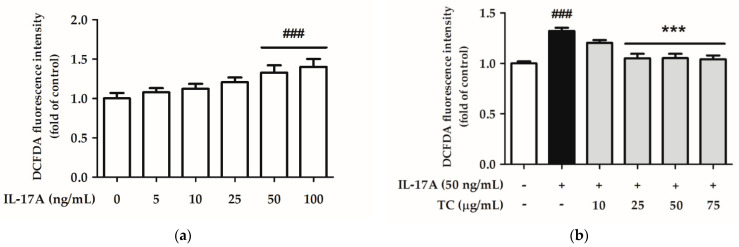
(**a**) IL-17A-induced and (**b**) TC inhibits IL-17A-induced intracellular oxidative stress in human keratinocytes. IL-17A at a concentration of 50 and 100 ng/mL significantly induced ROS generation, and TC suppressed IL-17A-induced ROS production in a dose-dependent manner in human keratinocytes. Significant differences between treated groups were evaluated. ^###^ *p* < 0.001 compared to the control group. *** *p* < 0.001 compared to the IL-17A-induced group.

The ROS levels generated after treatment with 50 and 100 ng/mL IL-17A increased by 1.29 and 1.40 times, respectively, compared to those in the control group, showing a significant difference (Figure 7). Therefore, an IL-17A induction dose of 50 ng/mL was selected for subsequent experiments.

Intracellular oxidative stress is related to the initiation of inflammatory and immunomodulatory pathways. Therefore, we speculated that TC inhibits IL-17A-induced ROS production. To test this hypothesis, HaCaT cells were co-treated with 50 ng/mL IL-17A and different concentrations of TC (0, 10, 25, 50, and 75 µg/mL) for 24 h. Subsequently, ROS levels were analyzed by DCFDA assay, followed by MTT assay to evaluate cell viability. Treatment with 50 ng/mL IL-17A alone successfully increased ROS levels, while co-treatment with concentrations of TC above 25 µg/mL significantly inhibited ROS generation compared with the IL-17A-induced group (Figure 7).

**Figure 8:**
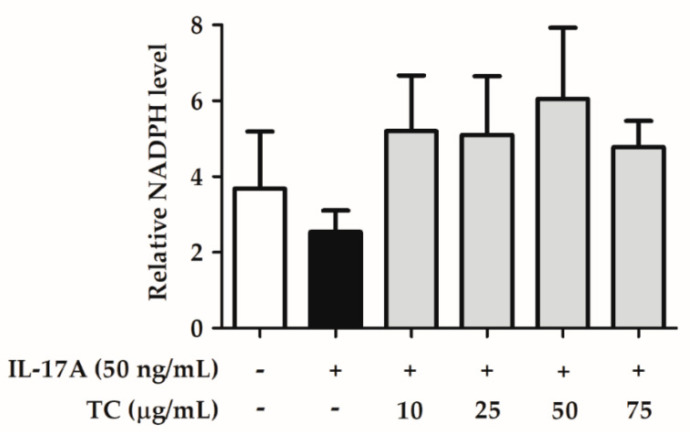
TC restores basal NADPH levels in IL-17A-treated human keratinocytes. TC could restore reduced NADPH levels upon IL-17A treatment in human keratinocytes.

Furthermore, cell lysates were collected and analyzed using an NADPH assay kit. The reduction in NADP^+^ generates NADPH, which inhibits the production of ROS. Figure 8 shows that IL-17A induction restrained NADPH generation, while co-application of IL-17A with TC restored the basal NADPH level. These results suggest that TC could inhibit IL-17A-induced intracellular oxidative stress by reducing ROS levels and increasing NADPH levels.

### 2.5. Influence of TC on the Activation of the Cell Proliferation Signaling Pathway

**Figure 9:**
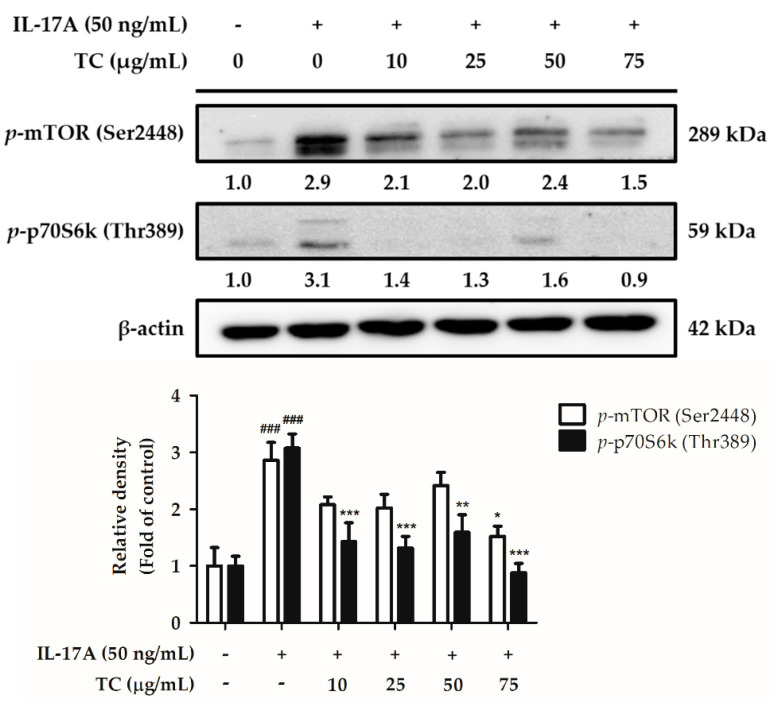
TC suppresses IL-17A-induced cell proliferation in human keratinocytes via the *p*-mTOR/*p*-p70S6k signaling pathway. TC could inhibit IL-17A-induced *p*-mTOR and *p*-p70S6k expression in human keratinocytes, thereby possibly suppressing excess activation of the cell proliferation pathway. Significant differences between treated groups were evaluated. ^###^ *p* < 0.001 compared to the control group. * *p* < 0.05; ** *p* < 0.01; *** *p* < 0.001 compared to the IL-17A-induced group.

Skin lesions in psoriasis patients are characterized by hyper-proliferation of keratinocytes. Therefore, we assumed that TC could inhibit the IL-17A-induced cell proliferation pathway. To test this hypothesis, HaCaT cells were co-treated with 50 ng/mL IL-17A and different concentrations of TC (0, 10, 25, 50, and 75 µg/mL) for 24 h. The cell lysates were analyzed by Western blotting to detect the expression of proteins involved in the mTOR/p70S6K signaling pathway. Interestingly, *p*-mTOR expression increased by 2.5 times in the IL-17A-treated group with respect to the control group, while treatment with TC inhibited IL-17A-induced *p*-mTOR expression. In particular, a TC concentration of 75 µg/mL significantly decreased (by 0.64 times) the relative density of p-mTOR with respect to that of the IL-17A-treated group. Moreover, the expression of p-p70S6K in IL-17A-treated cells was 3.1 times higher than that in the control group, while treatment with a TC concentration above 10 µg/mL notably reduced IL-17A-induced p-p70S6K expression (Figure 9). These results showed that TC could inhibit cell proliferation by regulating the expression of proteins implied in the mTOR/p70S6K signaling pathway.

### 2.6. Influence of TC on the Inflammatory Signaling Pathway

**Figure 10:**
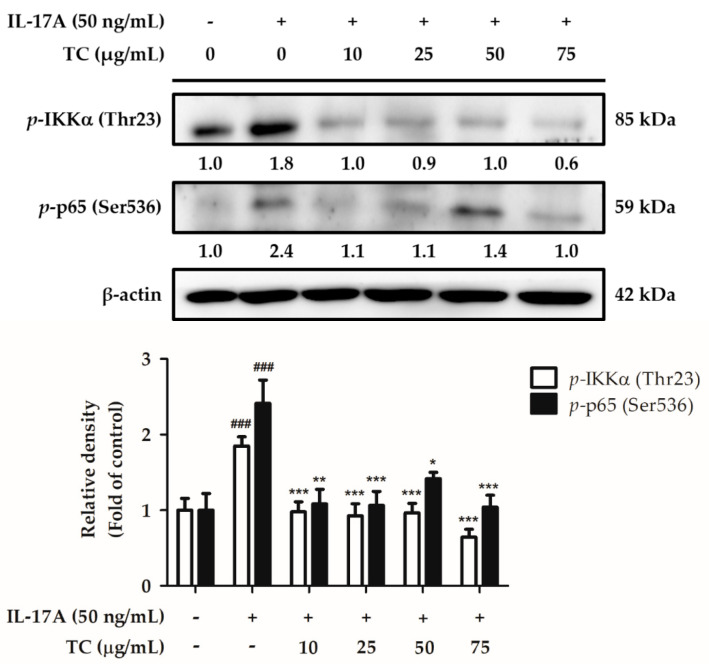
TC inhibits IL-17A-induced expression of pro-inflammatory proteins related to the NF-κB signaling pathway in human keratinocytes. TC could inhibit IL-17A-induced NF-κB expression in human keratinocytes, thereby possibly suppressing inflammatory responses. Significant differences between treated groups were evaluated. ^###^ *p* < 0.001 compared to the control group. * *p* < 0.05; ** *p* < 0.01; *** *p* < 0.001 compared to the IL-17A-induced group.

**Figure 11:**
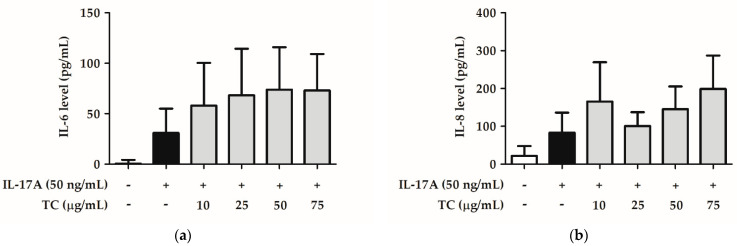
Effect of TC on IL-6 and IL-8 levels in IL-17A-treated human keratinocytes. (**a**) IL-6 level. and (**b**) IL-8 level. TC did not significantly reduce IL-17A-induced IL-6 and IL-8 levels in human keratinocytes.

Psoriasis is a disease associated with sustained inflammation; therefore, we hypothesized that TC could reduce the IL-17A-induced skin inflammatory signaling pathway. To test this hypothesis, HaCaT cells were co-treated with 50 ng/mL IL-17A and different concentrations of TC (0, 10, 25, 50, and 75 µg/mL) for 24 h. The cell lysates and the cell supernatants were separately analyzed by Western blotting and ELISA to investigate the activation status of the NF-κB signaling pathway. IL-17A treatment induced a significantly increased (1.8 fold) expression of *p*-IKKα, while co-treatment with a TC concentration above 10 µg/mL significantly reduced *p*-IKKα expression (Figure 10). Moreover, IL-17A treatment induced a significant increase in *p*-p65 expression by 2.4 fold, whereas a TC concentration of 75 µg/mL inhibited the increase by 1.0 times compared to the IL-17A-treated group. Next, we measured the levels of cytokines downstream of the NF-κB pathway, in particular IL-6 and IL-8 levels (Figure 11). IL-17A treatment increased the expression of these cytokines; however, TC failed to repress the enhanced expression. Altogether, these results indicated that TC could significantly inhibit IL-17A-induced *p*-IKKα and *p*-p65 expression but did not affect IL-6 and IL-8 levels.

## 3. Discussion

Oxidative stress is one of the key factors in the formation of psoriasis or skin disorders. We evaluated the antioxidant ability of TC using DPPH scavenging, reducing power, and H_2_O_2_ inhibition assays. The results reported in Figure 1, Figure 2 and Figure 3 showed that TC is a potent antioxidant. Our results are consistent with those of other studies [32,33]. Furthermore, to investigate the efficacy of TC against intracellular oxidative stress, a DCFDA assay and an NADPH assay were performed. Cellular esterases break down the DCFDA reagent into DCFH, which is then converted into DCF by reacting with ROS, thereby producing fluorescence [34]. Kumar et al. demonstrated that ethanol extracts of *T. camphoratus* mycelia suppressed ethanol-induced ROS levels in the HepG2 human hepatoma cell line [35]. Moreover, Li et al. discovered that *T. camphoratus* fruiting body extract suppressed Th17 cell differentiation and imiquimod-induced psoriasis-like symptoms in C57BL/6 mice [36]. Similarly, our results showed that TC concentrations above 25 µg/mL significantly inhibited IL-17A-induced ROS production. This effect might depend on the reduction in NADP^+^ to generate NADPH, which inhibits the production of ROS. Interestingly, Hsieh et al. found that methylantcinate A isolated from TC inhibited NADPH oxidase activity [37]. This finding is in line with our results, showing that TC restored IL-17A-reduced NADPH levels and inhibited ROS production.

The IL-23/IL-17 axis is one of the driving factors of psoriasis pathogenesis as the cytokine IL-23 activates the differentiation of Th17 cells, while IL-17 stimulates inflammatory cascades leading to the emergence of psoriasis. Subsequently, the cytokine IL-17A produced by Th17 cells binds to IL-17RA on keratinocytes and triggers ROS production by stimulating the activity of the NADPH oxidase [4]. This enzyme catalyzes the conversion of NADPH to NADP^+^ yielding ROS, including superoxide and hydrogen peroxide, as byproducts, thereby affecting cell growth, migration, and metabolism [38]. Overproduction of ROS activates pro-inflammatory and innate immune responses, mediated by the NF-κB, MAPK, and AKT/mTOR signaling pathways [39].

*Taiwanofungus camphoratus*, a medicinal fungus endemic to Taiwan, mainly consists of a dominant mycelial structure, is involved in nutrient absorption, and produces fruiting bodies for multiplication. Although the fruiting body of TC contains more abundant active compounds than the mycelium, the fruiting body grows very slowly in the wild environment and usually takes several months to cultivate. Due to the scarcity and high harvest cost, mycelium fermented products are usually used in traditional medicine and research. Fermented cultivation is an alternative production method with stable quality, which possesses the advantages of a short culture time period and high feasibility of scale-up production [29,40]. Sesame oil was used as the antifoam agent and growth promotion agent undergoing co-fermented cultivation with TC in our study. The stimulation of cell growth by oils was attributed to the partial incorporation of lipids in the cell membrane and facilitated the uptake of nutrients from the medium [41]. Sesamin, the major lignan present in sesame, is known for its antioxidative properties [42]. In a previous study, sesamin not only reduced intracellular ROS production after UV irradiation but also inhibited NF-κB translocation to decrease inflammation [43]. EK100 is also the bioactive compound derived from TC. EK100 ameliorated the epidermal thickness and the overexpression of IL-6 and NF-κB in chronic UV exposure hairless mice [28]. Both sesamin and EK100 have potential antioxidant and anti-inflammatory effects on the skin. It may contribute to the amelioration activity of TC on IL-17A-induced damage in keratinocytes.

Both IL-17A and ROS were found to stimulate the activation of the NF-κB and PI3K/AKT/mTOR signaling pathways involved in the pathogenesis of psoriasis. The NF-κB pathway participates in the interaction between keratinocytes and T cells, and thus, triggers sustained inflammatory and immune responses [44]. The IKK phosphorylation leads to the dissociation of the IKK/NF-κB complex, leading to NF-κB translocation into the nucleus, followed by increased production of inflammation-related proteins [45]. Downstream of the NF-κB pathway, IL-6, IL-8, and TNF-α lead to chronic inflammation and inhibit the expression of regulatory T cell proteins. In addition, the expression of angiogenetic factors such as G-CSF, GM-CSF, and the adhesion factor ICAM-1 is promoted by the NF-κB pathway, resulting in immune cell infiltration [46,47,48]. Furthermore, the skin of psoriatic lesions exhibits increased NF-κB expression compared to the perilesional skin area [49]. According to previous reports, suppression of the NF-κB pathway may hamper psoriasis initiation; thus, in this study, we investigated the inhibitory effect of TC on the NF-κB pathway, as a sustained inflammatory response may lead to psoriasis development. Notably, TC is rich in triterpenoids and polysaccharides, and previous reports have demonstrated that the active ingredients of TC exert anti-inflammatory properties. In particular, Shie et al. revealed that dimethoxy-5-methyl-1,3-benzodioxole, isolated from TC, inhibited lipopolysaccharide-induced activation of the NF-κB signaling pathway in RAW264.7 cells [50]. Moreover, methylantcinate B, a triterpenoid isolated from the fruiting body of *T. camphoratus*, suppressed NF-κB activation induced by the bacterial pathogen *Helicobacter pylori* in gastric epithelial cells [51]. EK100 is the active compound in TC, and it inhibited ultraviolet B-induced expression of IL-6 and NF-κB markers in skin of nude mice [26]. Similarly, the present study showed that TC decreased IL-17A-induced *p*-IKKα and *p*-p65 expression, pointing at the ability of TC to exert anti-inflammatory effects.

**Figure 12:**
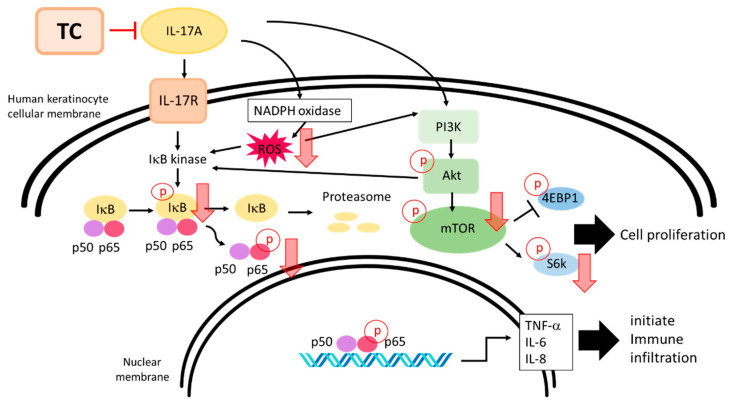
Schematic diagram illustrating the inhibitory effects of TC on IL-17A-induced inflammation and proliferation in human keratinocytes.

IL-17A and ROS were demonstrated to trigger the phosphorylation of PI3K and AKT, leading to the phosphorylation of mTOR [52]. Phosphorylated mTOR activates the downstream molecule p70S6K and inhibits 4E-BP1, resulting in the regulation of cellular functions such as proliferation, activation, and protein synthesis [53]. In addition, several studies based on psoriatic plaques and psoriatic tissue biopsies have shown that increased activation of the mTOR signaling cascade causes increased phosphorylation of mTOR and p70S6K. As suggested by these reports, inhibition of the *p*-mTOR/*p*-p70S6K pathway may reduce cell-proliferation-dependent psoriatic lesions. Thus, in this study, we examined the inhibitory effect of TC on the *p*-mTOR/*p*-p70S6K pathway. Skin lesions in psoriasis patients are characterized by hyper-proliferation of keratinocytes. Notably, Endo et al. indicated that the AKT/mTOR/p70S6K signaling pathway is involved in cell proliferation, cell invasion, and cell motility [14]. Moreover, Varshney and Saini discovered that IL-17A could induce mTOR and p70S6K expression in HaCaT cells [52]. Interestingly, the results of the present study suggest that TC may reduce IL-17A-induced expression of phosphorylated mTOR and p70S6K, demonstrating that TC can inhibit cell proliferation by regulating the mTOR/p70S6K signaling pathway (Figure 12). The effect of TC on psoriasis may be further confirmed by testing in an animal model.

## 4. Materials and Methods

### 4.1. Chemicals and Reagents

Bradford reagent was purchased from Bio-Rad Laboratories (Hercules, CA, USA). 1,1-diphenyl-2-picrylhydrazyl (DPPH), 2′,7′-dichlorofluorescein diacetate (DCFDA), leupeptin, and phenylmethylsulfonyl fluoride were purchased from Sigma-Aldrich (St. Louis, MO, USA). Recombinant human IL-17A was purchased from PEPROTECH (Cranbury, NJ, USA). Sodium dodecyl sulfate (SDS), MTT, Tris, and Tween20 were obtained from USB Corporation (Cleveland, OH, USA). Fetal bovine serum (FBS), penicillin-streptomycin, trypsin-EDTA, and Dulbecco’s modified Eagle medium (DMEM) were purchased from Gibco-Invitrogen (Carlsbad, CA, USA). Anti-*p*-IKKα, anti-*p*-mTOR, and anti-*p*-p65 antibodies were purchased from Genetex (Beverly, MA, USA). Anti-*p*-p70S6k and anti-β-actin antibodies were purchased from arigo (Hsinchu, Taiwan). All other chemicals used in this study were of reagent grade.

### 4.2. Preparation of Fermented TC Extract

*Taiwanofungus camphoratus* mycelia were kindly provided by Professor Kuo from the Department of Chinese Pharmaceutical Sciences and Chinese Medicine Resources, China Medical University, Taiwan. *T. camphoratus* mycelium and sesame oil are co-fermented for ten days to obtain the *T. camphoratus* mycelium fermented product. Five kilograms of the fermented *T. camphoratus* mycelia product were subjected to methanol extraction; after vacuum concentration, the extract was collected, and its composition was analyzed as previously described [25,28].; the TC yield was approximately 37%.

### 4.3. High-Performance Liquid Chromatography

The HPLC system consisted of a Waters 2695 Alliance Separations Module HPLC system (Milford, MA, USA) with a diode array detector (Waters 2996 Photodiode Array Detector). HPLC separation was performed on a reversed phase column (XTrra^®^ RP18 Column, 4.6 mm I.D. × 250 mm, 5 μm) thermostatted at 25 °C, using a mobile phase that consisted of water and acetonitrile. The gradient program was set as follows: 60:40–5:95 (0–50 min); 5:95 (50–60 min). The flow rate was 1 mL/min and the wavelength was 280 nm. Each injection volume was 10 μL.

### 4.4. Antioxidant Assays

#### 4.4.1. DPPH Radical Scavenging Assay

The assay was modified from a previous study [54]. Briefly, 100 μL/well of sample or vehicle (as control) was added to a 96-well plate. The sample and blank solutions were mixed with DPPH and methanol, respectively. After 30 min of reaction, the absorbance values were measured at 517 nm. The scavenging rate was calculated as previously described [54].

#### 4.4.2. Reducing Power Assay

The assay was modified from Berker et al. [55]. One hundred microliters of the sample, PBS buffer, and potassium ferricyanide solution were mixed in an Eppendorf tube and incubated at 50 °C for 20 min, then placed at 4 °C for 5 min. Trichloroacetic acid solution was added for precipitation, and 100 µL/well of supernatant was placed in a 96-well plate. Then, 100 µL of sterilized water and 10 µL of FeCl_3_ solution were added. The reaction was incubated at room temperature for 20 min, after which the absorbance was read at 700 nm. The reducing power was calculated as previously described [56].

#### 4.4.3. H_2_O_2_ Inhibition Assay

The assay was modified from a previous study [56]. A standard curve was set with diluted concentrations of an H_2_O_2_ solution. Next, 100 µL/well of sample or vehicle (as control) was added to a 96-well plate, then sample and blank solutions were mixed with H_2_O_2_ solution and PBS, respectively. After 10 min of reaction, the absorbance was measured at 230 nm, the values were compared with the H_2_O_2_ standard curve, and the H_2_O_2_ inhibition rate was calculated.

### 4.5. Cell Culture and MTT Assay

Human keratinocytes (HaCaT cells) were purchased from the AddexBio Technologies (San Diego, California, USA). Cells were incubated in an incubator containing 5% CO_2_ at 37 °C and grown in DMEM supplemented with 10% FBS, 100 U/mL penicillin, and 100 µg/mL streptomycin [34]. HaCaT cells were cultured at 2 × 10^5^ cells/well in 24-well plates for 24 h. Then, the medium was removed and replaced with the sample solution. After incubation for 24 h, the sample solution was aspirated, and cells were incubated in 300 µL of 0.5 mg/mL MTT for 3 h. Next, the MTT solution was removed, and 500 µL of isopropanol was added. The absorbance was measured at 570 nm, and cell viability was calculated as previously described [57].

### 4.6. DCFDA Assay

HaCaT cells were cultured at 2 × 10^5^ cells/well in 24-well plates for 24 h, and then the medium was removed and replaced with the sample solution. After 24 h, the sample solution was aspirated, and cells were washed with PBS buffer, treated with 1 mL/well of a 10-µM DCFDA solution, and incubated for 30 min. Subsequently, the DCFDA solution was removed and replaced with 500 µL of PBS buffer. The excitation and emission wavelengths were set up at 488 nm and 520 nm, respectively, to detect the fluorescence of ROS.

### 4.7. Western Blotting

The assay was modified from a previous study [34]. Briefly, samples were loaded into the sample wells of an SDS-polyacrylamide gel electrophoresis (PAGE) gel and separated by gel electrophoresis at a voltage of 80 V for 30 min in the stacking gel and a voltage of 120 V for 70 min in the running gel. Subsequently, a polyvinylidene fluoride (PVDF) membrane was activated with methanol. Bands on the gel were transferred to the PVDF membrane, which was then submerged into Tris-buffered saline with Tween 20 supplemented with 5% skim milk (5% MTBST) for 30 min and washed with TBST three times. Next, the membrane was incubated with primary antibodies at 4 °C overnight. The primary antibodies were then removed and the membrane was rinsed with TBST three times and then incubated with secondary antibodies at room temperature for 3–4 h. Thereafter, the secondary antibodies were discarded and the membrane was treated with an ECL substrate. Finally, protein density on the membrane was detected using an ImageQuant LAS 4000 instrument (GE Healthcare, Chicago, IL, USA) and analyzed with the Multi Gauge software (Fujifilm, Tokyo, Japan).

### 4.8. NADPH Assay

This assay was carried out with an NADPH Assay kit (Abcam, Cambridge, UK) according to the manufacturer’s instructions. Briefly, an NADPH standard solution was prepared to quantify the NADPH content of samples. Then, 50 µL/well of cell lysates were added to a 96-well plate, mixed with 50 µL/well of NADPH reaction mixture and incubated for 2 h at room temperature. The absorbance was measured at 460 nm, and values were compared with the standard curve. The NADPH level was calculated by normalizing the measured NADPH concentration by the protein content of the cell lysate.

### 4.9. ELISA

ELISA was carried out following the protocols of the Human IL-6 (interleukin 6) ELISA kit (Elabscience^Ⓡ^, Houston, TX, USA) and the Human IL-8 (interleukin 8) ELISA kit (Elabscience^Ⓡ^), with modifications. Briefly, 100 µL/well of standard working solution and sample were added to a primary antibody-coated 96-well plate and incubated at 37 °C for 90 min. Afterward, the liquid was removed from each well, and 100 µL of biotinylated detection Ab working solution was added. The plate was then incubated at 37 °C for 60 min. Subsequently, each well was washed with wash buffer three times; next, 100 µL of horseradish peroxidase (HRP) conjugate working solution was added, and the plate was incubated at 37 °C for 30 min. After washing with wash buffer five times, 90 µL of substrate reagent was added, and the plate was incubated at 37 °C for 15 min. Finally, 50 µL of stop solution was added to stop the reaction and the optical density of each well was determined. The absorbance was measured at 450 nm, and the absorbance values were compared with the standard curve. The cytokine level was calculated by normalizing the measured cytokine concentration by the protein content of the cell supernatant.

### 4.10. Statistical Analysis

All experimental data were analyzed using the GraphPad Prism software package version 5.0 (San Diego, CA, USA) and ImageJ. Values are presented as mean ± SD. Statistical differences were determined using one-way ANOVA and Tukey’s post-hoc test. Results with *p* < 0.05 were considered statistically significant.

## 5. Conclusions

In this study we found that extracts of *Taiwanofungus camphoratus* mycelia (TC) exhibited a remarkable antioxidant ability and inhibited IL-17A-induced intracellular oxidative stress. Subsequently, we demonstrated that TC could regulate IL-17A-induced inflammation and cell proliferation via the mTOR/p70S6K and NF-κB signaling pathways and, thus, may play a role in the pathogenesis of psoriasis (Figure 12). Based on our results, TC may be applied as an anti-psoriasis agent, as well as for the treatment of other inflammatory-related diseases.

## Data Availability

Not applicable.
